# Computational assessment of groundwater salinity distribution within coastal multi-aquifers of Bangladesh

**DOI:** 10.1038/s41598-022-15104-x

**Published:** 2022-07-01

**Authors:** Mehdi Jamei, Masoud Karbasi, Anurag Malik, Laith Abualigah, Abu Reza Md Towfiqul Islam, Zaher Mundher Yaseen

**Affiliations:** 1 Faculty of Engineering, Shohadaye Hoveizeh Campus of Technology, Shahid Chamran University of Ahvaz, Dashte Azadegan, Iran; 2 Water Engineering Department, Faculty of Agriculture, University of Zanjan, Zanjan, Iran; 3 Punjab Agricultural University, Regional Research Station, Bathinda, Punjab India; 4 Faculty of Information Technology, Middle East University, Amman, Jordan; 5 Department of Disaster Management, Begum Rokeya University, Rangpur, 5400 Bangladesh; 6 New Era and Development in Civil Engineering Research Group, Scientific Research Center, Al-Ayen University, Thi-Qar, 64001 Iraq; 7 Department of Earth Sciences and Environment, Faculty of Science and Technology, Universiti Kebangsaan Malaysia, Bangi, 43600 Selangor Malaysia

**Keywords:** Environmental sciences, Environmental impact, Computational science, Marine chemistry

## Abstract

The rising salinity trend in the country’s coastal groundwater has reached an alarming rate due to unplanned use of groundwater in agriculture and seawater seeping into the underground due to sea-level rise caused by global warming. Therefore, assessing salinity is crucial for the status of safe groundwater in coastal aquifers. In this research, a rigorous hybrid neurocomputing approach comprised of an Adaptive Neuro-Fuzzy Inference System (ANFIS) hybridized with a new meta-heuristic optimization algorithm, namely Aquila optimization (AO) and the Boruta-Random forest feature selection (FS) was developed for estimating the salinity of multi-aquifers in coastal regions of Bangladesh. In this regard, 539 data samples, including ten water quality indices, were collected to provide the predictive model. Moreover, the individual ANFIS, Slime Mould Algorithm (SMA), and Ant Colony Optimization for Continuous Domains (ACOR) coupled with ANFIS (i.e., ANFIS-SMA and ANFIS-ACOR) and LASSO regression (Lasso-Reg) schemes were examined to compare with the primary model. Several goodness-of-fit indices, such as correlation coefficient (R), the root mean squared error (RMSE), and Kling-Gupta efficiency (KGE) were used to validate the robustness of the predictive models. Here, the Boruta-Random Forest (B-RF), as a new robust tree-based FS, was adopted to identify the most significant candidate inputs and effective input combinations to reduce the computational cost and time of the modeling. The outcomes of four selected input combinations ascertained that the ANFIS-OA regarding the best accuracy in terms of (R = 0.9450, RMSE = 1.1253 ppm, and KGE = 0.9146) outperformed the ANFIS-SMA (R = 0.9406, RMSE = 1.1534 ppm, and KGE = 0.8793), ANFIS-ACOR (R = 0.9402, RMSE = 1.1388 ppm, and KGE = 0.8653), Lasso-Reg (R = 0.9358), and ANFIS (R = 0.9306) models. Besides, the first candidate input combination (C1) by three inputs, including Cl^−^ (mg/l), Mg^2+^ (mg/l), Na^+^ (mg/l), yielded the best accuracy among all alternatives, implying the role importance of (B-RF) feature selection. Finally, the spatial salinity distribution assessment in the study area ascertained the high predictability potential of the ANFIS-OA hybrid with B-RF feature selection compared to other paradigms. The most important novelty of this research is using a robust framework comprised of the non-linear data filtering technique and a new hybrid neuro-computing approach, which can be considered as a reliable tool to assess water salinity in coastal aquifers.

## Introduction

In many places of the world, groundwater is the most crucial water source for economic development and human survival^1^; it is the typical source of drinking water in many parts of the world. Groundwater can be regarded as a renewable natural resource because it can be refilled continually in most circumstances^2^. More than 2.5 billion people globally depend on groundwater for water supply and to keep many key terrestrial ecosystems alive^3^. In many parts of the world, excess water exploration due to increasing water demand has threatened their long-term viability^4,5^. The encroachment of seawater into coastal aquifers and the removal of water from coastal aquifers have caused erratic changes in the quality of groundwater and water flow patterns.

Recently, the growing demand for groundwater resources has subjected such natural resources to more strain than ever before^6,7^. In unconfined coastal aquifers, the most typical problem is groundwater salinization, mainly when excessive groundwater pumping reduces the piezometric head^8^. Confining layers that partly separate seawater from groundwater and the vadose zone has complicated the hydrogeological processes in confined and semi-confined coastal aquifers^9^. As a result, seawater can easily intrude into semi-confined coastal aquifers. The problem of groundwater depression has been documented in various cases worldwide^10–12^. The increasing level of salt accumulation in both plants and soil has significantly increased groundwater salinity, which has negatively impacted ecological health, the economic advancement of residents, and the productivity of coastal crops. Increased salinity also hurts drinking water quality, thereby jeopardizing human health^13,14^. Furthermore, groundwater salinization results in an increased quantity of salt in the root zone, which creates an osmotic impact on plants, forcing them to expend more energy to absorb water from the soil, thereby limiting their ability to develop^15^.

Another issue is that excessive groundwater extraction and poor management have established local or regional groundwater depressions in several regions^16^. Prolonged over-extraction of groundwater can result in depression, as seen in the North China Plain, where several towns, including Cangzhou, Dezhou, and Tianjin, were in severe water depression ^17^. Excessive groundwater extraction from aquifers in Dhaka, Bangladesh, has also been reported to cause an exponential fall in groundwater levels around the city and water quality-related issues^18^. Several wells were installed in Tripoli, Northwest Libya, to pump groundwater in the city; this act caused a sharp decline in groundwater level; a further construction of a groundwater depression caused the limited discharge of freshwater to the ocean^19^. Groundwater formation may also result in geological risks, such as ground cracks and land subsidence. In China, excessive groundwater extraction in some representative locations has been reported to cause geological problems due to prolonged over-extraction consistently distributed with groundwater depression^20^.

Based on the reported literature, the salinity in groundwater is stochastic, and this is because several parameters affect its concentration and magnitude. Those parameters include upward instruction from deep aquifers^21^, evaporation rate from soil^22^, irrigation saline water, and wastewater infiltration^23^. Hence, understanding the actual mechanism of groundwater salinization and the affected sources is essential for water resources management and sustainability^24,25^.

Worth mentioning, Electrical conductivity (EC) is used to explain the salinity of water; the concentration of dissolved salts is a metric to determine the EC of groundwater^26^. In a thoroughly prepared groundwater sample, EC is generally tested by creating an electric current between the two electrodes of a salinometer. This approach is point-based and analyses the EC of the tested groundwater samples. Although this process is accurate, the preparation step makes it time-consuming to perform over a large area. In the local setting, direct current resistivity methods examine EC distribution. In this method, the potential field is determined using two additional electrodes; a current is created and delivered into the ground by point electrodes^27^. However, this is a slow procedure that cannot be applied on a regional scale. Previous studies on mapping groundwater salinity based on a regional scale using the feasibility of remote sensing data have been conducted remarkably^28–31^. However, the main concept of using geographical information system data for mapping the salinity is to estimate the salinity for unknown data points using the interpolation technique within a defined range of measured data points. The sensing technique has its merits. It is quick and straightforward to apply; however, it is connected with significant error calculation and is proportional to the square of the distance between data points^32^. Also, it does not consider the sample's distribution in high salinity areas. Hence, introducing new technology, such as computer aid models for solving this complex and significant natural issue, is one of the prioritized research topics in water and geo-science.

The ability to predict groundwater quality is crucial to comprehending its evolution trend^33,34^. This is especially useful for determining groundwater quality in the groundwater depression cone zone. Numerical modeling and statistical prediction methods are available for predicting groundwater quality. However, machine learning (ML) models have been recently reported as a new method that substantially impacts groundwater quality modeling^35,36^. As a known data analysis method, ML models can automate the framework of an analytical model. Artificial intelligence (AI) is based on the idea that machines can learn from data, recognize patterns, and make decisions with minimal human interaction^37,38^. The ability of ML models to model groundwater salinity has been demonstrated via the establishment of a linear or non-linear relationship between water salinity and its control parameters (such as water table, evaporation, and distance to saltwater bodies) and using those relationships for the prediction of water salinity for regions with unavailable data points^39,40^. Various versions of ML models have been reported in the literature, such as artificial neural network (ANN)^41–45^, support vector machine (SVM)^46–48^, adaptive neuro-fuzzy inference system (ANFIS)^49,50^, ensemble ML models^38,51,52^, group method of data handling (GMDH)^53^, and Gaussian process scheme^54^. The significant limitations associated with predictive ML models (1) the need for adequate input variables to explain the target data that may not be available everywhere^55,56^, (2) the influence of well excessive pumping^57,58^, (3) the reliability of the learning process of the predictive model where essential hyperparameters are optimized^59,60^, (4) coupled ML models where a pre-processing technique was integrated for data time series decomposition^61,62^. The ML model was adopted based on the inspiration of developing a new hybrid model for the ANFIS model. In groundwater quality modeling, hybrid ANFIS showed a promising result^63,64^.

Due to the highly non-linear relationships between input predictors and water quality targets in coastal aquifers, using a scientific-based strategy to determine the optimal candidate input combinations for feeding the ML methods is very important. It has received less attention in the previous literature. In most previous research, regardless of the behavior of the data, a certain number of possible input combinations have been examined using the ML methods, and superior results have been presented. However, selecting specific combinations without a scientific basis may increase the uncertainty and decrease the accuracy of the outcomes. This motivated this study focuses on three significant aspects. The aims of the current investigation, novel predictive models, were developed based on the hybridization of the ANFIS model with new nature-inspired optimization algorithms (i.e., Aquila optimization) for groundwater salinity prediction. The second aim is to inspect the highly influential predictors using the newly explored feature selection algorithm, Boruta-Random Forest. The outcomes of the primary model were examined with standalone ANFIS, ANFIS-SMA, ANFIS-ACOR, and Lasso-Reg approaches. Finally, the current research was adopted on a significant case study, “coastal areal of Bangladesh,” where the groundwater salinity is a vital issue for that region. The current research can provide an essential vision for introducing a reliable computer aid model.

## Materials and methods

### Study area description

With approximately 24,000 km2, the coastal regions are primarily low-lying areas in the southern portion of Bangladesh along the Bay of Bengal (BoB). Diverse geomorphic characteristics of the coastal district include the deep funnel-shaped structures of the BoB's northern landfill. Tidal fluctuation is prevalent in most river systems from the coastal area, causing fluctuations of 2–4 m. Groundwater pollution is caused by a rise in the relative sea level, rapid population increase, a poor drainage system, salinity intrusion, and other factors. Aquaculture and agricultural activities are the primary sources of income in the coastal regions. Due to rising soil salinity, aquaculture, particularly shrimp culture, has expanded while paddy crop production has decreased^65^. The typical coastal climatic system is characterized by a warm and tropical environment dominated by the BoB's southwest monsoonal flow. The average annual precipitation and temperature (June–September) are 2000–2500 mm and 25 °C, respectively.

The Bengal Basin's coastal area started in the late Holocene to the Recent Age^66^. The study area formed the basin's deeper portion throughout the Holocene age. The lithology of this area is varied, with coarse-to fine-grained sandstone and peat soil combined with silty clay^65^. Each sediment layer containing groundwater comprises coarse-grain sand, fine-grained silt, and clay^67^. The coastal region's hydrogeology comprises unconsolidated Quaternary alluvial sediments that are covered by a thick (3 to 7 m) silty-clay layer. The shallow aquifer depth ranged from 10 to 50 m, with salt concentrations ranging from 1500 mg/L to 2000 mg/L. Rainwater collecting, especially during the monsoon season, is another viable source of freshwater. The people rely on salinity-rich rivers, channels, and fishponds for their water supply^68^. Furthermore, rainwater recharges the shallow aquifer during the dry winter season, which is invaded excessively^69^.

There are three types of aquifers^70^. The upper shallow aquifer is located northwest of the coastal area, with a thickness varying from a few meters to 60 m. Second, the shallow aquifer, which has a thickness ranging from 10 m to more than 100 m^71^, contains saltwater pockets, particularly abundant in coastal and estuarine flooded areas. Third, there is a deep aquifer with a thickness of more than 200 m and various features in the southern portion of the coastal zone.

The water in this region's aquifer is frequently replenished by rainfall, rivers, and channels^72^. The groundwater is significantly depleted during the dry and monsoon seasons and subsequently refilled. Groundwater flow may have aided the saltwater infiltration into the water-bearing strata. The parent rock impacts the water chemistry, and numerous types of minerals found in the aquifer regulate the water quality. According to the available geological data, the aquifers in this area are either unconfined or semi-confined.

### Data description and sampling technique

**Figure 1:**
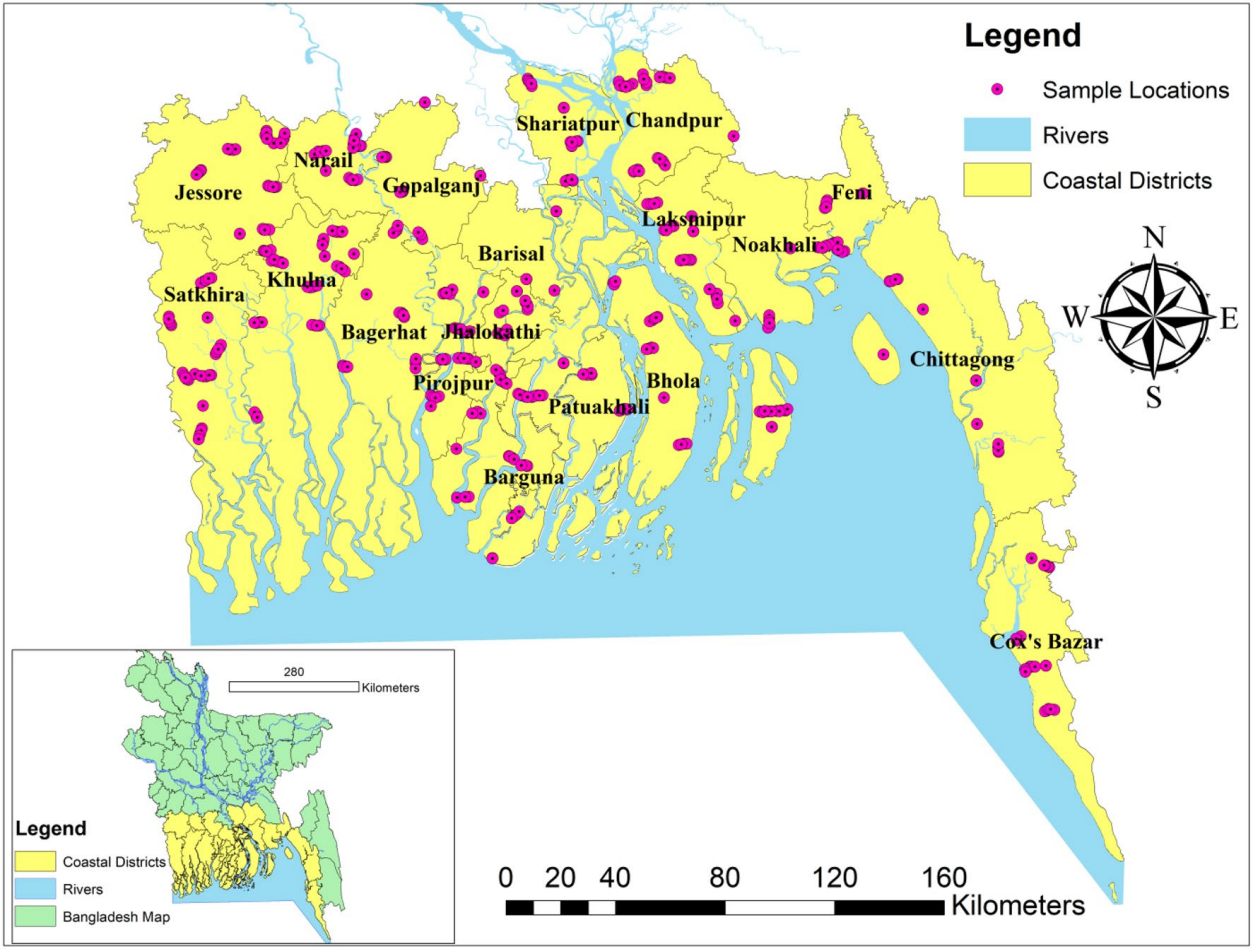
Location of sampling point in area study during the wet season; map of coastal regions of Bangladesh.

For using machine learning methods, a large dataset is required. The datasets used in this study came from^65^ and^73^, and the sampling design and analytical procedures are described below. During the wet season, 539 groundwater samples were collected from three campaigns between 2015 and 2017. Each sample was assigned an ID number, and coordinates were confirmed using a portable GPS device^74^, as shown in Fig. 1. Before collecting the sample from the tube well, the groundwater was pumped for at least 10 min to remove any standing water.

The pumping of the sampling tube well was continued until the pH and electrical conductivity (EC) were both steady. The samples were collected in prewashed high-density polypropylene (HDPP) bottles^66,75^. It is worth noting that each station collected two sets of replicated samples. The samples were collected and filtered using 0.45 m membranes from MF-Millipore™ in the United States. The samples' HDPP bottles were kept at 4 °C in a more excellent box and subsequently sent to the laboratory for further analysis. While EC and pH were measured using portable pH/EC meters (Hanna HI 9811–5).

A field survey was used to measure groundwater depth and salinity during the wet seasons. Ion chromatography with Dionex ICS-90 was used to determine cations (Ca^2+^, Mg^2+^, Na^+^, K^+^) and anions (Cl^–^, HCO_3_^–^, NO_3_^–^, PO_4_^2–^, SO_4_^2–^, and F^–^). Five standard solutions (1, 5, 10, 15, and 20 mg/L) were employed during the calibration process. A conventional laboratory process and quality control checks were used to provide quality assurance. Three replicated samples were obtained simultaneously to ensure that the test results were accurate by cross-checking with a qualified laboratory. The ion charge balance error (ICBE), which was used to determine accuracy, ranged from 2.63 to 8.62 percent, with a mean of 8.24 percent, well within the permissible limit of 10%. Table 1 lists the descriptive statistics of datasets used in the salinity assessment of the multi-aquifers in coastal regions of Bangladesh. As can be seen, the maximum kurtosis values were owing to the PO_4_^–2^ (mg/l), K^+^ (mg/l), and Ca^2+^ (mg/l).

**Table 1:** descriptive statistics of all groundwater quality parameters for modeling the salinity using three hybrid ANFIS models and the Lasso-Reg approach.

Variables	Minimum	Maximum	Mean	Std. D	COV (%)	Skewness	Kurtosis
Depth (m)	18	336	113.1	62.84	55.56	1.957	3.141
pH	6.2	8.8	7.336	0.4449	6.065	0.0911	0.943
Ca^2+^ (mg/l)	0.2511	902.4	89.82	111	123.6	3.479	17.15
Mg^2+^ (mg/l)	0.0681	718.9	107.1	131.6	122.9	1.889	3.419
Na^+^ (mg/l)	0.2	4211	722.5	836.8	115.8	1.295	0.789
K^+^ (mg/l)	0.252	176.8	10.33	16.38	158.5	5.806	43.54
HCO_3_^−^ (mg/l)	24.4	823.5	221.7	119.3	53.81	1.123	1.91
SO_4_^2−^(mg/l)	0	2926	22.87	141.1	617.2	16.9	336
PO_4_^−2^ (mg/l)	0.03	57.7	1.359	3.498	257.4	10.34	142.6
Cl^−^ (mg/l)	2.1	16,250	2054	2713	132.1	1.928	4.64
Salinity (ppt)	0	25.5	3.05	3.661	120.0	1.769	4.073

### Boruta-Random forest feature selection

The selection of features is critical in implementing machine learning algorithms^76^. Boruta is an algorithm for feature selection. More precisely, it acts as a wrapper algorithm for Random Forest. This algorithm is named after a monster from Slavic folklore who inhabited pine trees. The stage of feature selection is critical in predictive modeling. This strategy is vital when a data set including several variables is provided for model construction. This is particularly true when the goal is to understand the mechanics behind the interest variable rather than merely build a high-prediction-accuracy black-box model. Boruta determines the Z-scores for each input predictor concerning the shadow property. The distribution of Z-score metrics reveals the essential characteristics of the predictors^77^. A minimal-optimal feature selection technique was used by ranking and residuals based on the Boruta-determined relevance criteria, followed by stepwise model development^78^.

1. To begin, it randomizes the input data set by making scrambled duplicates of all features (shadow features).
2. Then, it trains a random forest classifier on the larger data set and evaluates the value of each feature using a feature importance measure (the default is Mean Decrease Accuracy), where greater equals more significant. The following Equation calculates the MDA:
  1 $$MDA=\frac{1}{{m}_{tree}}\sum_{m=1}^{{m}_{tree}}\frac{\sum_{t\in OOB}I({y}_{t}=f({x}_{t}))-\sum_{t\in OOB}I({y}_{t}=f({x}_{t}^{n}))}{\left|OOB\right|}$$
  where OOB denotes out-of-bag (i.e., the prediction error for each of the training trials aggregated by bootstrap), whereas $({y}_{t}=f({x}_{t}))$ and $({y}_{t}=f({x}_{t}))$ denote the predicted values before and after permutation, respectively. Additionally, $I()$ denotes the indicator function.
3. Each iteration determines if a genuine feature is more essential than the best of its shadow features (i.e., whether the feature has a higher Z score than the shadow features' maximum Z score) and continually eliminates features thought to be very irrelevant. The Z-score is computed as follows:
  2 $$Z-score=\frac{MDA}{std}$$
  where $std$ is the standard deviation of accuracy losses, and then the maximum Z-score for duplicate attributes was computed (MZSA).
4. If Z-scores are less than MZSA, the inputs are tagged "unimportant" and separated permanently until inputs with Z-scores more than MZSA are designated "Confirmed".
5. Finally, the method terminates when all features have been validated or rejected or the required number of random forest iterations reached.

### Lasso regression

Robert Tibshirani coined the term LASSO in 1996^79^. It is a robust approach that accomplishes two primary tasks: regularization and feature selection. The Lasso approach constrains certain of the model parameters' absolute values. The total must be less than a preset value (upper bound). To do this, the approach employs a shrinkage (or regularization) procedure in which it penalizes the coefficients of regression variables, thereby shrinking them to zero^80^. Incorporating a penalty item into linear regression may dramatically reduce the variance of a model by effectively shrinking the coefficient estimates, particularly in models with high-dimension predictors^81^. The optimized objective function of Lasso Regression (Lasso-Reg) is as follows:

3 $$\sum_{i=1}^{n}{\left({y}_{i}-{\beta }_{0}-\sum_{j=1}^{p}{\beta }_{j}{x}_{ij}\right)}^{2}+\Gamma \sum_{j=1}^{p}\left|{\beta }_{j}\right|$$

where ${\beta }_{0}$ denotes the Lasso-Reg shift and ${\beta }_{j}$ denotes the ${x}_{ij}$ coefficients. In this relation, $\Gamma$ is a regulation parameter, which means that if its value is equal to zero, the model becomes a normal regression, and all variables will be present, and if its value increases, the number of independent variables in the model will decrease. The determination of the value for this parameter is usually done by the cross-validation method^80^.

### Adaptive neuro-fuzzy inference system (ANFIS)

The ANFIS technique is based on a knowledge-based mix of fuzzy inference systems (FIS) and artificial neural networks (ANN). The FIS can generate IF–THEN fuzzy rules from fuzzy sets with an adequate membership function (MF) to represent human thought, but its capabilities are restricted to adaptive learning^82^. While ANNs are capable of adaptive learning for decision-making, they cannot explain how the choice was formed. Thus, incorporating adaptive learning capabilities from ANNs into the IF–THEN fuzzy rules in FIS structures becomes more powerful and may be utilized to tackle complicated engineering or non-engineering issues in various applications^83^.

**Figure 2:**
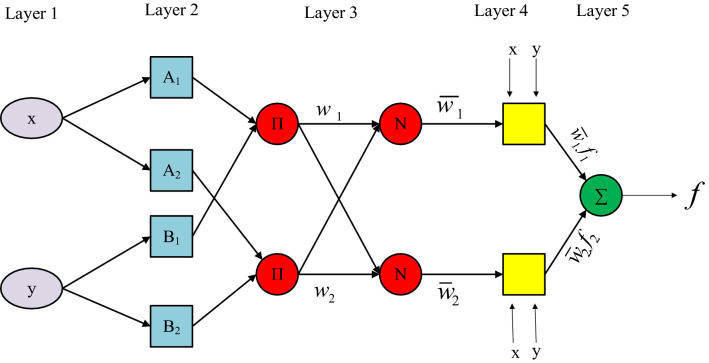
Structure of the ANFIS model.

The ANFIS model is used in this work due to its high capacity for learning and superior performance^84^. The ANFIS model is structured in two parts: antecedent and consequent. To keep things simple, the ANFIS structure is configured with two inputs, x, and y, as seen in Fig. 2. The ANFIS model is composed of five levels structurally. Each level has a distinct role, detailed below^85^.

1. Layer 1 (Fuzzification layer): This layer accepts discrete input values and gives them membership functions.
  4 $${O}_{1,i}=\mu {A}_{i}\left(x\right) i=\mathrm{1,2}$$
  5 $${O}_{1,i}=\mu {B}_{i-2}\left(y\right) i=\mathrm{3,4}$$
  The input nodes are represented by $x$ and $y$. The linguistic variables are denoted by $A$ and $B$. ${A}_{i}\left(x\right)$ and ${B}_{i-2}\left(y\right)$ are node membership functions.
2. Layer 2 (Rule layer): Each rule's firing strength is created in this layer using the product operation.
  6 $${O}_{2,i}={w}_{i}=\mu {A}_{i}\left(x\right)\mu {B}_{i}\left(y\right) i=\mathrm{1,2}$$
  where ${w}_{i}$ denotes each node's output.
3. Layer 3 (Normalization layer): This phase normalizes the firing strength of each rule to the total firing strength.
  7 $${O}_{3,i}={\overline{w} }_{l}=\frac{{w}_{i}}{{w}_{i}+{w}_{2}} i=\mathrm{1,2}$$
4. Layer 4 (Defuzzification layer): This layer takes as normalized input values and their corresponding parameters (${p}_{i}$, ${q}_{i}$, and ${r}_{i}$). The defuzzified values are returned after combining these arguments.
  8 $${O}_{4,i}={\overline{w} }_{l}{f}_{i}={\overline{w} }_{l}\left({p}_{i}x+{q}_{i}y+{r}_{i}\right)$$
5. Layer 5 (Output layer): The final output is a weighted average of the output from each rule.
  9 $${O}_{5,i}=\sum {\overline{w} }_{l}{f}_{i}=\frac{\sum {\overline{w} }_{l}{f}_{i}}{\sum {w}_{i}} i=\mathrm{1,2}$$

The most significant notion in ANFIS is determining the number of membership functions. This may be regarded as a clustering problem; consequently, the FCM is employed to create a limited number of fuzzy rules. Bezdek invented the FCM in 1984^86^. Each data point in the FCM method belongs to one of the clusters with a membership value that varies from zero to one. The FCM may be obtained by optimizing the objective function below^87^.

10 $${J}_{FCM}={\sum }_{i=1}^{c}{\sum }_{j=1}^{n}{u}_{ij}^{m}{\Vert {x}_{j}-{v}_{i}\Vert }^{2}$$

where ${u}_{ij}$ is membership degree, c is the total number of clusters, $m$ is a constant value, $\Vert {x}_{j}-{v}_{i}\Vert$ is Euclidean distance of ${x}_{j}$ from ${i}$th cluster center ${v}_{i}$. In the FCM technique, the cluster center and membership degree may be computed using the following Eqs. ^87^:

11 $${v}_{i}=\frac{{\sum }_{j-1}^{n}{u}_{ij}{x}_{j}}{{\sum }_{j-1}^{n}{u}_{ij}} i=\mathrm{1,2},\dots ,c$$

12 $${u}_{ij}=\frac{1}{{\sum }_{k=1}^{c}{\left(\frac{\Vert {x}_{j}-{v}_{i}\Vert }{\Vert {x}_{j}-{v}_{k}\Vert }\right)}^{\frac{2}{m-1}}}$$

### Aquila optimizer (AO)

Aquila Optimizer (AO) is a novel nature-inspired algorithm proposed by Abualigah et al. in^88^. The following subsections explain how the AO models these processes.

AO simulates Aquila's hunting behavior by demonstrating the actions taken at each hunt stage^89^. The AO algorithm's optimization processes are divided into four categories. The following is a mathematical model of the AO.

#### Step 1: Expanded exploration

The Aquila accepts the prey area and chooses the best hunting area by high soar with the vertical stoop in the first searching technique (*X*_*1*_). Equation (3) represents this behavior mathematically^90^.

13 $${X}_{1}\left(t+1\right)=Xbest\left(t\right)*\left(1-\frac{t}{T}\right)+({X}_{m}\left(t\right)-Xbest\left(t\right)*rand)$$

where X1(t + 1) is the solution created by the first search method for the next iteration of t. (*X*_*1*_). The best-obtained solution at the tth iteration is Xbest(t), representing the prey's approximate location. $(1-\frac{t}{T}$) is used to control the number of iterations in the expanded search (exploration). A random value between 0 and 1 is called rand. The current iteration and the maximum number of iterations are represented by t and T, respectively.

14 $${X}_{M}\left(t\right)=\frac{1}{N}\sum_{i=1}^{N}{X}_{i}\left(t\right), \forall j=1\dots \dots .Dim$$

where Dim is the problem's dimension size and N is the number of possible solutions in the population.

#### Step 2: Narrowed exploration

When the prey area is discovered from a high vantage point, the Aquila circles above the target prey prepares the land, and then attacks. Equation (15) represents this behavior mathematically^91^.

15 $${X}_{2}\left(t+1\right)=Xbest\left(t\right)*Levy\left(D\right)+{X}_{R}\left(t\right)+\left(y-x\right)*rand$$

where X_2_(t + 1) is the solution created by the second search method (X_2_) for the next iteration of t. Levy(D) is the levy flight distribution function calculated using Eq. (16). At the ith iteration, XR(t) is a random solution taken in [1 N].

16 $$Levy\left(D\right)=s*\frac{u*\sigma }{{|\nu |}^{\frac{1}{\beta }}}$$

where s is a constant value of 0.01, u, and $\nu$ are random integers between 0 and 1, and $\sigma$ is a value calculated by using Eq. (17).

17 $$\sigma =(\frac{\Gamma\left(1+\beta \right)*sine(\frac{\pi \beta }{2})}{\Gamma\left(\frac{1+\beta }{2}\right)*\beta *{2}^{(\frac{\beta -1}{2})}})$$

where $\beta$ is a constant with a value of 1.5; in Eq. (15), the spiral shape in the search is represented by y and x, which are computed as follows.

Where

18 $$y=r*\mathrm{cos}\left(\theta \right), x=r*sine\left(\theta \right), r={r}_{1}+U.{D}_{1}$$

19 $$\theta =-\omega *{D}_{1}*{\theta }_{1}, {\theta }_{1}=\frac{3*\pi }{2}$$

**Figure 3:**
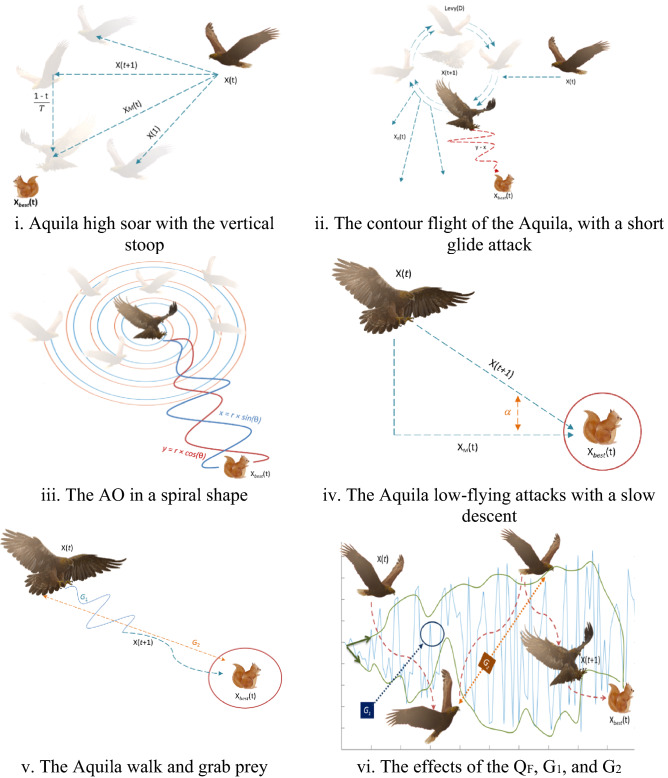
the schematic view of the OA algorithm.

For a fixed number of search cycles, r_1_ takes a value between 1 and 20, and U is a small value set to 0.00565. D_1_ is an array of integer numbers ranging from 1 to the search space length (Dim), and $\omega$ is a small value set to 0.005. Figure 3 depicts the AO's behavior in a spiral shape.

#### Step 3: Expanded exploitation

When the prey area is precisely defined and the Aquila is ready to land and attack, the third technique (X_3_) is used. Equation (20) represents this behavior mathematically^92^.

20 $${X}_{3}\left(t+1\right)=\left(Xbest\left(t\right)-{X}_{M}\left(t\right)\right)* \alpha -rand+\left(\left(UBj-LBj\right)*rand+LBj\right)*\delta$$

where X_3_(t + 1) is the solution of the next iteration of t, which is generated by the third search method (X_3_). Xbest(t) refers to the approximate location of the prey until ith iteration, and X_M_(t) denotes to the mean value of the current solution at tth iteration, which is calculated using Eq. (14). "rand" is a random value between 0 and 1. $\alpha$ and $\delta$ are the exploitation adjustment parameters fixed in this paper to a small value (please refer to Table 3).

#### Step 4: Narrowed exploitation

When the Aquila approaches the prey in the fourth technique (X_4_), the Aquila attacks the prey over land based on their stochastic movements. Equation (21) represents this behavior mathematically^89^.

21 $${X}_{4}\left(t\right)=QF* Xbest\left(t\right)-\left({G}_{1}*X\left(t\right)*rand\right)-{G}_{2}*Levy\left(D\right)+rand*{G}_{1}$$

where X4(t + 1) is the solution of the fourth search method's (X4) for the next iteration of t, the quality function (Q_F_) is used to balance the search strategies and is calculated using Eq. (21). G_1_ refers to various AO motions that are used to track the prey during the elope and are generated using Eq. (21). G_2_ shows decreasing values from 2 to 0, which represent the AO's flight slope as it follows the prey during the elope from the first (1) to the last (t) location, as calculated using Eq. (21). The current solution at the tth iteration is X(t).

22 $${Q}_{F}\left(t\right)= {t}^{(\frac{2*rand-1}{{(1-T)}^{2}})}, {G}_{1=2*rand-1}, {G}_{2}=2*(1-\frac{t}{T})$$

The quality function value at the tth iteration is Q_F_(t), and the random value between 0 and 1 is rand. The current iteration and the maximum number of iterations are represented by t and T, respectively. The levy flight distribution function computed using Equation is Levy(D) (6). The effects of the Q_F_, G_1_, and G_2_ on the AO's behavior are shown in Fig. 3. The Pseudo-code of the AO is detailed in Algorithm 1.
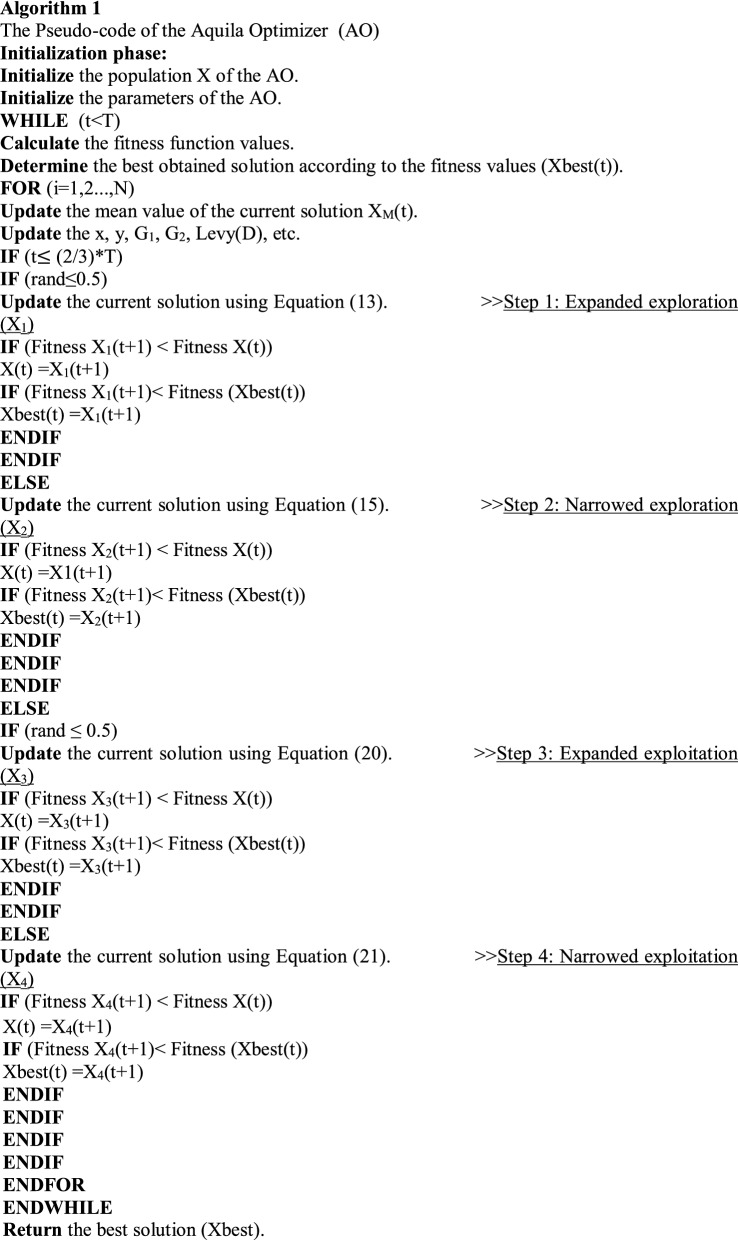


### Slime mould algorithm (SMA)

The physarum polycephalum is frequently described in conjunction with the slime mould. Slime mould is so termed because it is classified as a fungus^93^.

**Figure 4:**
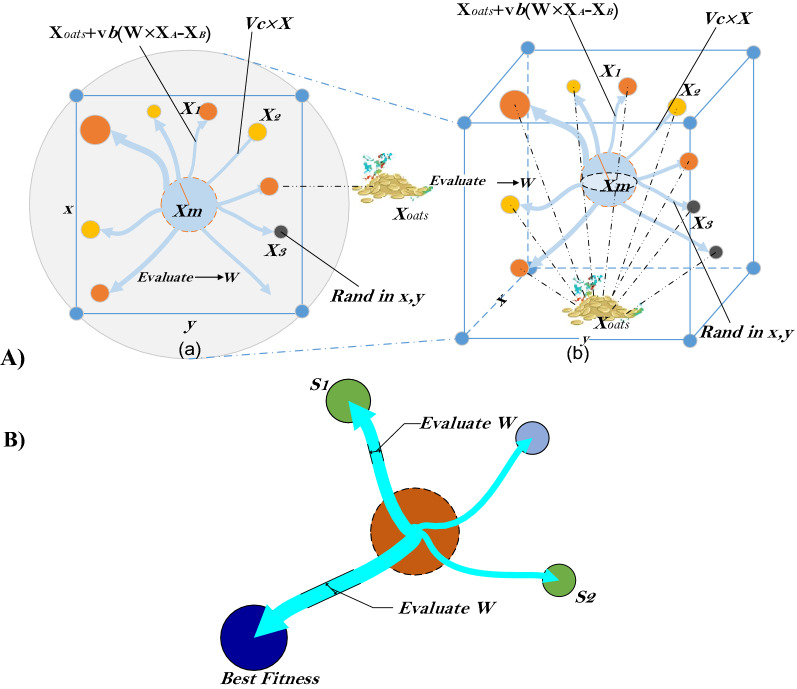
SMA algorithm stages; (**A**) Potential positions (**B**) Process of the fitness function.

- *Approach food*
  The following equations model depicts the SMA function. To replicate the constriction approach, the model equations are introduced:
  23 $$\overrightarrow{X\left(t+1\right)}=\left\{\begin{array}{c}\overrightarrow{{X}_{b}\left(t\right)}+\overrightarrow{vb}\cdot \left(\overrightarrow{W}\cdot \overrightarrow{{X}_{A}\left(t\right)}-\overrightarrow{{X}_{B}\left(t\right)}\right), r<p\\ \overrightarrow{vc}\cdot \overrightarrow{X\left(t\right)}, r\ge p\end{array}\right.$$
  where $\overrightarrow{vb}$ is a parameter utilized in $\left[-a,a\right]$, $\overrightarrow{vc}$ a parameter values changes from 1 to 0. $t$ is the *t*_*th*_ iteration,$\overrightarrow{{X}_{b}}$ iso the individual position of the current best,$\overrightarrow{X}$ is the position of the current solution, $\overrightarrow{{X}_{A}}$ and $\overrightarrow{{X}_{B}}$ are two solutions selected randomly, $\overrightarrow{W}$ are the weight of the current solution^94^. The $p$ value is dertermined as follows:
  24 $$p=\mathrm{tanh}\left|S\left(i\right)-DF\right|$$
  where $i\in 1, 2, 3,.\dots ,n$, $S\left(i\right)$ is the fitness function of the current solution, $DF$ is the best-obtained fitness value. The $\overrightarrow{vb}$ is dertermined as follows:
  25 $$\overrightarrow{vb}=\left[-a,a\right], a=\mathrm{arctanh}(-\left(\frac{t}{\mathrm{max}\_iter}\right)+1)$$
  The $\overrightarrow{W}$ is determined as follows:
  26 $$\overrightarrow{W(SmellIndex(i))}=\left\{\begin{array}{c}1+r\cdot \mathit{log}\left(\frac{bF-S\left(i\right)}{bF-wF}+1\right),condition \\ 1-r\cdot \mathit{log}\left(\frac{bF-S\left(i\right)}{bF-wF}+1\right), others\end{array}\right.$$
  $$SmellIndex=sort(S)$$
  where *r* is a random value in $\left[\mathrm{0,1}\right]$, $bF$ is the best-obtained fitness value, $wF$ is the worst obtained fitness value, $SmellIndex$ is sorted fitness value. Figure 4a shows the impacts of potential positions^95^.
- *Wrap food*
  When the food product is pleased to stretch to a place where the food quantity is weak, the priority of that region decreases, causing researchers to shift their attention to other regions of food availability that are not as significant as the food product. Figure 4b depicts the rule for assessing slime mould fitness values.
  The mathematical representation for updating positions is given as follows:
  27 $$\overrightarrow{{X}^{*}}=\left\{\begin{array}{c}rand\cdot \left(UB-LB\right)+LB,rand<z \\ \overrightarrow{{X}_{b}\left(t\right)}+\overrightarrow{vb}\cdot \left(W\cdot \overrightarrow{{X}_{A}\left(t\right)}-\overrightarrow{{X}_{B}\left(t\right)}\right),r<p \\ \overrightarrow{vc}\cdot \overrightarrow{X\left(t\right)}, r\ge p\end{array}\right.$$
  where $LB$ and $UB$ are the lower bound and upper boundaries $rand$ and $r$ are random values in [0,1], z is a parameter value in [0, 0.1].
- *Grabble food*

$\overrightarrow{vb}$ is an area of random numbers in $[-a,a]$. The $\overrightarrow{vc}$ is given in [-1,1]. Although slime mould received a better feed supply, it would still spread organic material for seeking other locations for an upper-class food supply rather than investing all of it in a single area to discover a more reliable supply of nutrition. The SMA algorithm's mechanism is depicted in Algorithm 2.
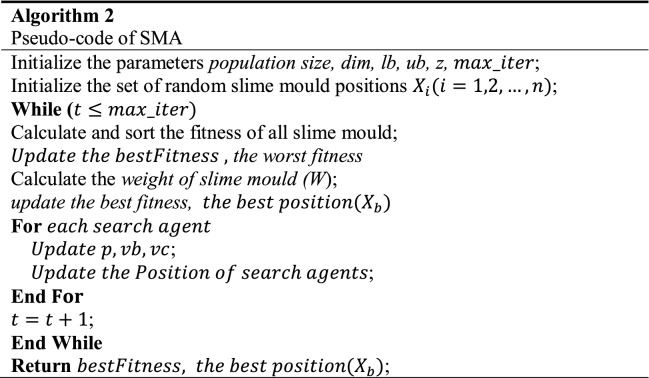


### Ant colony optimization (ACO)

Ant colony optimization (ACO), invented by Dorigo^96^, is a multi-agent approach used to tackle optimization issues. This method is based on observational data of real ants seeking food. Ants are small social insects in colonies and cooperate to ensure the colony's survival. While hunting for food, the ants inspect their surroundings and mark them with pheromones, which the colony's other members can follow. When ants locate a food supply, they attempt to nurture it by transporting it back to the colony via the nearest root^97^. The ACO algorithm uses a discrete structure to determine the answer. The concept of discrete structure in ACO means that each decision variable in the defined interval is divided into a certain number of states. By discretizing the space of variables, there is a limit to the algorithm, reducing accuracy. In this regard, ACO generalization to continuous space was considered. If the decision variable space is assumed to be continuous, the algorithm will move in the R space of real numbers. The ACOR algorithm performs spatial integration in decision variables using a probability density function (PDF). Sosha and Dorigo proposed using a Gaussian function to create such a structure^98^. A one-dimensional Gaussian function cannot produce a maximum of several points, whereas using a Gaussian kernel function, the sum of the weights of several single Gaussian functions, can perform such a task. The following Equation defines the weighted sum of 1-D Gaussian functions:

28 $${G}^{i}\left(x\right)={\sum }_{l=1}^{k}{w}_{l}{g}_{l}^{i}\left(x\right)={\sum }_{l=1}^{k}{w}_{l}\frac{1}{{\sigma }_{l}^{i}\sqrt{2\pi }}{e}^{-\frac{{\left(x-{x}_{l}^{i}\right)}^{2}}{2{{\sigma }_{l}^{i}}^{2}}}$$

where $i$ is the dimension of the problem, k denotes the total number of best solutions in the solution repository. ${w}_{l}$ is the weight that each solution receives based on its rank and it can be calculated using following Eq. ^98^:

29 $${w}_{l}=\frac{1}{\sqrt{2\pi qk}}{e}^{\frac{-{\left({r}_{l}-1\right)}^{2}}{{q}^{2}{k}^{2}}}$$

${r}_{l}$ is rank of solutions. The $q$ parameter (Intensification Factor) affects the minimum and maximum limits of ${w}_{l}$. When $q$ is small, the solutions with the highest rankings are highly preferred. Equation 29 is used to compute the elements of the weight vector x. Following that, the sampling process is finished in two steps. The first step is to select one of the Gaussian functions that comprise the Gaussian kernel PDF. The following formula expresses the probability of selecting the ${l}$th Gaussian function^98^:

30 $${p}_{l}=\frac{{w}_{l}^{l}}{{\sum }_{r=1}^{k}{w}_{r}^{r}}$$

The chosen Gaussian function is sampled in the second phase. This can be accomplished by employing a random number generator capable of producing random numbers based on a parameterized normal distribution. The standard deviation of a normal distribution PDF is ${\sigma }_{l}^{i}$ and it is determined using the following Equation:

31 $${\sigma }_{l}^{i}=\xi {\sum }_{e=1}^{k}\frac{\left|{S}_{e}^{i}-{S}_{l}^{i}\right|}{k-1}$$

where $e$ denotes the iteration and $k$ denotes the solution's number in the solution archive. $\xi$ (Deviation-Distance Ratio) is a parameter that controls the convergence speed. The algorithm's convergence speed decreases with increasing $\xi$. Solution ${S}_{l}^{i}$ has rank $l$ and ${S}_{e}^{i}$ is the solution in the current iteration^98^. The ACO_R_ method refines and regenerates the solution archive in each iteration by adding m new solutions ($k\to k+m$) and then removing the worst $m$ solutions ($k+m\to k$) in order to maintain the solution archive's size constant (negative and positive update). As a result of the changes to the solutions recorded in the solution archive, the pheromone for each iteration is increased in optimized paths that do not improve the objective function. Thus, the ACO_R_ algorithm finds the optimal solution. For more simplicity, hereafter, the ACO_R_ is called ACO.

### Model performance evaluation

To assess the prediction performances of the various models, correlation coefficient (R), the root mean squared error (RMSE), Kling-Gupta efficiency (KGE) (Gupta et al., 2009), Willmott’s agreement Index (I_A_) (Willmott, 1982), Relative absolute error (RAE), Legate and McCabe’s Index (E_LM_) (Legates and McCabe, 2013) and coefficient of uncertainty with 95 confidence level (${U}_{95\%}$) were utilized^99–101^.

32 $$R=\frac{\sum_{\mathrm{i}=1}^{\mathrm{N}}\left({Salinity}_{o,i }- \overline{{Salinity }_{o }}\right) \left({Salinity}_{p,i} - \overline{{Salinity }_{p}}\right) }{\sqrt{\sum_{\mathrm{i}=1}^{\mathrm{N}}({{Salinity}_{o,i }- \overline{{Salinity }_{o }})}^{2} \sum_{\mathrm{i}=1}^{\mathrm{N}}({{Salinity}_{p,i} - \overline{{Salinity }_{p}})}^{2} }}$$

33 $$RMSE=\sqrt{\frac{1}{N} \sum_{i=1}^{N}({Salinity}_{o,i }- {Salinity}_{p,i}{)}^{2}}$$

34 $$KGE = 1 - \sqrt {(R - 1)^{2} + (StD_{p} /StD_{o} - 1)^{2} + (\overline{{Salinity_{p} }} /\overline{{Salinity_{o } }} - 1)^{2} }$$

35 $${I}_{A}=1-\frac{{\sum }_{i=1}^{N}{\left({Salinity}_{o,i}-{Salinity}_{p,i}\right)}^{2}}{{\sum }_{i=1}^{N}{\left(\left|{Salinity}_{o,i}-{\overline{Salinity} }_{o}\right|+\left|{Salinity}_{o,i}-{\overline{Salinity} }_{o}\right|\right)}^{2}}$$

36 $$RAE = \frac{{\mathop \sum \nolimits_{i = 1}^{N} \left| {Salinity_{o,i} - Salinity_{p,i} } \right|}}{{\mathop \sum \nolimits_{i = 1}^{N} \left| {Salinity_{o,i} - \overline{{Salinity_{p,i} }} } \right|}}$$

37 $${U}_{95\%}=1.96\sqrt{{SD}_{e}^{2}+{RMSE}^{2}}$$

38 $${E}_{LM}=1-\frac{{\sum }_{i=1}^{N}{\left({Salinity}_{o,i}-{Salinity}_{p,i}\right)}^{2}}{{\sum }_{i=1}^{N}{\left({Salinity}_{o,i}- \overline{{Salinity }_{o }}\right)}^{2}}$$

where the predicted and actual values of salinity are ${Salinity}_{p,i}$ and ${Salinity}_{o,i}$, respectively. $\overline{{Salinity }_{p}}$ is the average of the predicted outcomes. $\overline{{Salinity }_{o}}$ is the average value of observed salinity values. The number of samples in the training or testing stage is denoted by $N$. ${SD}_{e}$ is the standard deviation of estimation error. $StD_{p}$ and $StD_{o}$ are the standard deviation of predicted and observed values, respectively. It should be emphasized that a model with R = 1, ${E}_{LM}$ = 1, RMSE = 0, RAE = 0, KGE = 1, ${I}_{A}=1$ and U_95%_ = 0 is a great model.

## Model development

### Feature selection procedure

**Figure 5:**
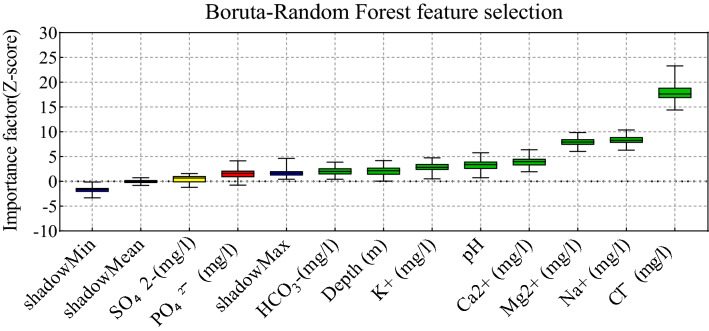
Feature selecting process using the Boruta-Random Forest method based on Z-score for all candidate input variables.

Here, the salinity of multi-aquifers in coastal regions of Bangladesh was modeled based on ten parameters, as reported in Table 1. As stated in literature, optimal input feature selection is one of the most important steps in developing an efficient predictive model with numerous features. On the other hand, linear regression-based methods such as correlation analysis and the best subset approaches may not correctly capture non-linear interactions between input and target parameters. Therefore, adopting an efficient strategy is inevitable. Recently, various strategies have been proposed that are appropriately able to detect non-linear aspects between data well. In the current research, the Boruta-Random forest feature selection^102^, was employed as a tree-based powerful feature selection to optimize the input combination and assess the critical degree of each input feature. The outcomes of the Boruta-Random forest feature selection based on the Z-score criterion are illustrated in Fig. 5, which implies the importance of each feature versus salinity. It can be included that Cl^-^, Na^+^, and Mg^2+^ features have a greater impact on modeling the salinity than the other parameter. Thus, those features were employed in all candidate input combinations. Also, the PH, K^+^, Depth, and HCO_3_^-^ were stood in the next rank and sequentially added to the significant features as the other candidate input combinations. In addition, the PO_4_^2−^ and SO_4_^2−^ regarding fewer Z-scores than the shadow max criterion were ignored to simulate the salinity of the multi-aquifers. The optimal candidate input combinations for more assessment via the predictive models were reported in Table 2.

**Table 2:** Selected scenarios of candidate input components for modeling the salinity of groundwater obtained via Burota-random forest feature selection.

Scenario	Variables	No
C 1	Cl^-^ (mg/l), Mg^2+^ (mg/l), Na^+^ (mg/l)	3
C 2	PH, Ca^2+^ (mg/l), Cl^-^ (mg/l), Mg^2+^ (mg/l), Na^+^ (mg/l), K^+^ (mg/l)	6
C 3	Depth (m), PH, Ca^2+^ (mg/l), Cl^−^ (mg/l), Mg^2+^ (mg/l), Na^+^ (mg/l), K^+^ (mg/l)	7
C 4	Depth (m), PH, Ca^2+^ (mg/l), Cl^−^ (mg/l), Mg^2+^ (mg/l), Na^+^ (mg/l), K^+^ (mg/l), HCO_3_^−^ (mg/l)	8

### model providing an optimal setting-parameter

**Figure 6:**
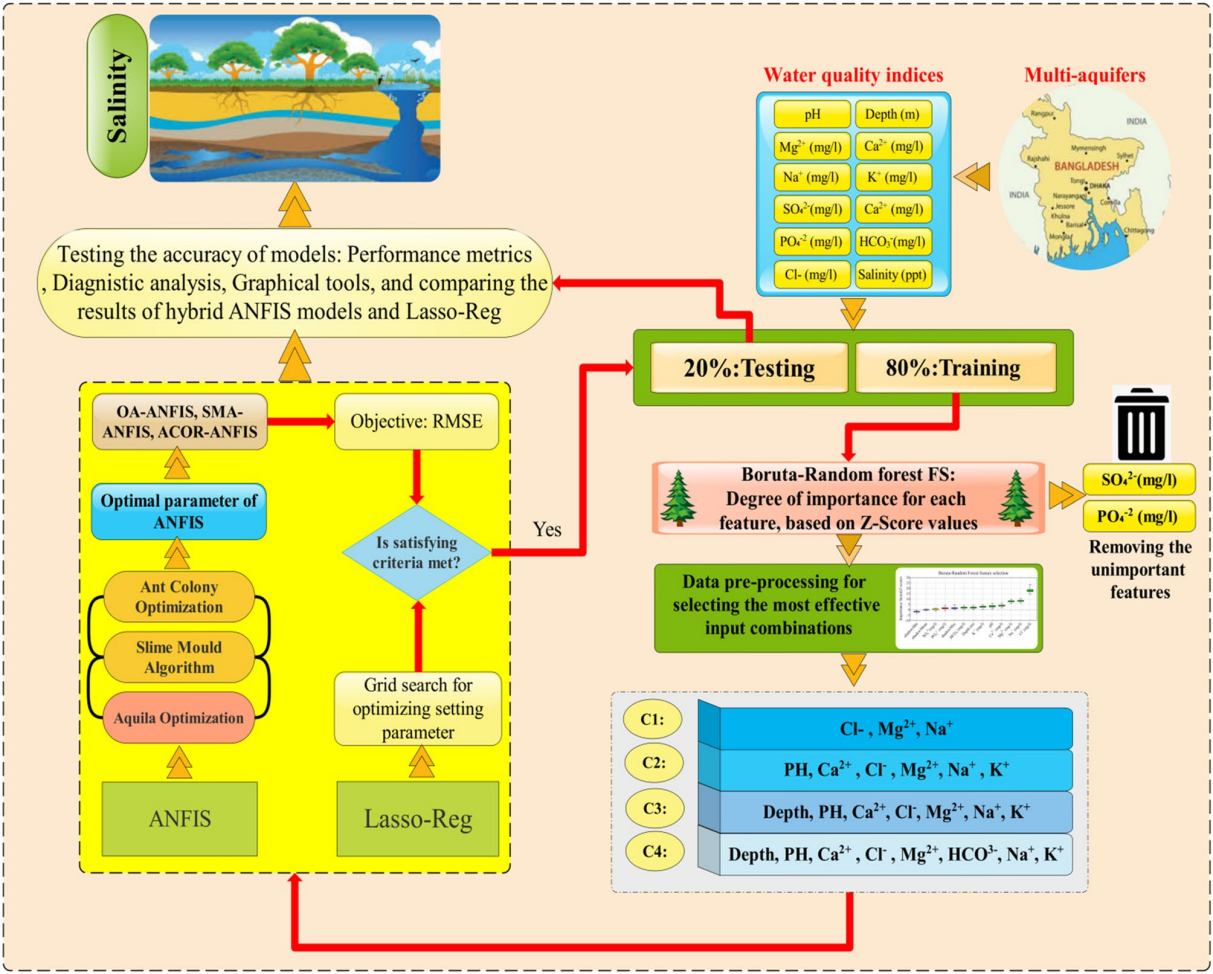
Workflow of salinity modeling based on a new ML-based hybrid strategy for multi-aquifers in coastal regions of Bangladesh.

Here, five efficient data-intelligent systems, including standalone ANFIS, OA-ANFIS, SMA-ANFIS, ACO-ANFIS, and Lasso-Reg, were examined to estimate the salinity of multi-aquifer in coastal regions of Bangladesh. The ANFIS and hybrid-ANFIS model were developed via Matlab 2019a, and the Lasso-Reg approach was adopted in Python platform 3.8 through the open-source sci-kit-learn library^103^, on a system with Intel Core (TM)i7-6700 CPU with 3.40 GHz. In order to optimize the most significant setting parameter of Lasso-Reg (i.e., $\Gamma$), the great search strategy was adopted in the range of $\Gamma \in$(0, 1). Besides, the standalone ANFIS approach was optimized based on a trial and error procedure by examing the cluster number in the range of (3–5). In the hybrid ANFIS model, membership functions of ANFIS were optimized using three algorithms (e.i., OA, SMA, and ACO) in popuse of the computational cost reduction and accuracy enhancement. Table 3 lists the optimum ANFIS setting parameters, critical values of the algorithm, and the Lasso-Reg tuning parameter. In this research, first, whole datasets (539 data points) are randomly divided into two subsets of training (80%) and testing (20%). Also, to avoid overfitting, the Monte Carlo approach has been employed with 500 runs, and the average results obtained from the Monte Carlo method have been considered. The workflow of salinity estimating the multi-aquifer in coastal regions of Bangladesh is demonstrated in Fig. 6.

**Table.3:** Optimal setting parameters owing to the standalone ANFIS model, optimization algorithm, and Lasso-Reg model.

Model/algorithm	Setting-tuning	Value
ANFIS	Epoch number	220
	Step Size Decrease	0.9
	Initial Step Size	0.01
	Step Size Increase	1.1
	Cluster number	3–5
SMA	Iteration	500
	Population	20
	z	0.03
ACO_R_	Iteration	500
	Population	20
	Intensification Factor	0.5
	Deviation-Distance Ratio	1
OA	Iteration	500
	Population	20
	$\alpha$	0.5
	$\delta$	0.5
	$\omega$	0.005
	$\beta$	1.5
Lasso-Reg	$\Gamma$	0–1

## Application results and analysis

The selection of the optimal input variables in modeling complex hydrological processes is very crucial. The present study utilized the Boruta-Random forest (B-RF) feature selection technique to nominate the most significant variables which affect the output and cast to develop the efficient data-intelligent models (standalone & hybrid). After the application of B-RF, four scenarios, i.e., C1 with 3 inputs, C2 with 6 inputs, C3 with 7 inputs, and C4 with 8 inputs (Table 2), were built to estimate groundwater's salinity in Bangladesh through standalone & hybrid machine learning (ML) models.

The results of the standalone and hybrid ML/data-intelligent models (i.e., ANFIS, ANFIS-OA, ANFIS-SMA, ANFIS-ACO, and Lasso-Reg) under C1 to C4 scenarios were evaluated based on seven statistical indicators including R, RMSE, RAE, E_LM_, KGE, I_A_, and U_95%_. Table 4 summaries the values of R, RMSE, RAE, E_LM_, KGE, I_A_, and U_95%_ during training and testing stages of various ML models under different scenarios. It was observed from Table 4 the standalone ANFIS model had poor performance among the other ML models based on statistical indicators in all four scenarios. The hybrids of ANFIS and Lasso-Reg models had improved performance over the ANFIS model in C1 to C4 scenarios. However, the superior performance of hybrid ANFIS-OA model was noted in terms of statistical indicators. The hybrid ANFIS-OA model had values of R = 0.9450, RMSE = 1.1253 ppt, RAE = 0.1878, E_LM_ = 0.8871, KGE = 0.9146, I_A_ = 0.9696 and U_95%_ = 3.0632 in scenario C1, R = 0.9374, RMSE = 1.1520 ppt, RAE = 0.2091, E_LM_ = 0.8774, KGE = 0.9100, I_A_ = 0.9671, and U_95%_ = 3.1931 in scenario C2, R = 0.9378, RMSE = 1.1685 ppt, RAE = 0.2083, E_LM_ = 0.8738, KGE = 0.8849, I_A_ = 0.9662, and U_95%_ = 3.2104 in scenario C3, and R = 0.9402, RMSE = 1.1489 ppt, RAE = 0.2073, E_LM_ = 0.8780, KGE = 0.9121, I_A_ = 0.9687, and U_95%_ = 3.1689 in scenario C4 during testing stage. The results indicate the significant improvement in the performance of ANFIS model while optimizing with OA algorithm than other algorithms. The comparison of employed ML models’ outcomes among the different scenarios are marked as C1 > C4 > C2, C3. Additionally, Habibi et al.^104^ proved the potential of hybrid ML model i.e., ANN-GA (artificial neural network-genetic algorithm) against the ANN, PSLR (partial least square regression), and DT (decision tree) models for soil salinity prediction in central Iran. Pouladi et al.^105^ predicted the soil salinity in Miandoab city of Iran by employing the MLP-FFA (multilayer perceptron-firefly algorithm) model using remote sensing and topography data. The outcomes of MLP-FFA model were compared with MLP model based on several statistical indices. Evaluation of results show that the MLP-FFA model had highest value of determination coefficient (R^2^ = 0.66), and lowest values of mean absolute error (MAE = 0.45 dS m^−1^), and RMSE = 0.54 dS m^−1^ than standalone MLP (R^2^ = 0.34, MAE = 0.54 dS m^−1^, RMSE = 0.67 dS m^−1^) model.

**Table 4:** The goodness of fit of the predictive model for estimation of the salinity of groundwater.

Models	Phase	R	RMSE	RAE	E_LM_	KGE	I_A_	U_95%_
**C1**								
ANFIS	Training	0.9596	1.0535	0.1646	0.9208	0.9428	0.9791	2.9218
	Testing	0.9306	1.2139	0.2131	0.8639	0.9222	0.9643	3.3699
ANFIS-OA	Training	0.9559	1.1183	0.1835	0.9108	0.9032	0.9760	3.0775
	**Testing**	**0.9450**	**1.1253**	**0.1878**	**0.8871**	**0.9146**	**0.9696**	**3.0632**
ANFIS-SMA	Training	0.9283	1.4083	0.2105	0.8585	0.9260	0.9632	3.9054
	**Testing**	**0.9406**	**1.1534**	**0.1896**	**0.8830**	**0.8793**	**0.9690**	**3.1632**
ANFIS-ACO	Training	0.9121	1.5501	0.2484	0.8286	0.8317	0.9488	4.2946
	Testing	0.9402	1.1688	0.2205	0.8802	0.8653	0.9656	3.1822
Lasso-Reg	Training	0.9013	1.6215	0.2348	0.8124	0.8602	0.9461	4.4971
	Testing	0.9358	1.1863	0.2091	0.8700	0.8552	0.9628	3.2766
**C2**								
ANFIS	Training	0.9761	0.8140	0.1529	0.9527	0.9662	0.9878	2.2577
	Testing	0.9090	1.3857	0.2238	0.8226	0.8965	0.9525	3.8451
ANFIS-OA	Training	0.9300	1.3851	0.2000	0.8631	0.9208	0.9639	3.8397
	**Testing**	**0.9374**	**1.1520**	**0.2091**	**0.8774**	**0.9100**	**0.9671**	**3.1931**
ANFIS-SMA	Training	0.9292	1.4446	0.2531	0.8511	0.9119	0.9632	4.0001
	Testing	0.9307	1.2801	0.2436	0.8486	0.8790	0.9626	3.5099
ANFIS-ACO	Training	0.9153	1.5079	0.2538	0.8378	0.8802	0.9544	4.1822
	Testing	0.9322	1.1930	0.2276	0.8685	0.9022	0.9642	3.3110
Lasso-Reg	Training	0.9149	1.5110	0.2477	0.8371	0.8776	0.9541	4.1907
	Testing	0.9331	1.1874	0.2232	0.8697	0.8982	0.9644	3.2936
**C3**								
ANFIS	Training	0.9740	0.8479	0.1604	0.9487	0.9633	0.9867	2.3516
	Testing	0.9205	1.3513	0.2412	0.8313	0.9073	0.9586	3.7540
ANFIS-OA	Training	0.9491	1.1871	0.2035	0.8994	0.9144	0.9730	3.2817
	**Testing**	**0.9378**	**1.1685**	**0.2083**	**0.8738**	**0.8849**	**0.9662**	**3.2104**
ANFIS-SMA	Training	0.9002	1.6635	0.2433	0.8025	0.8861	0.9476	4.6038
	Testing	0.9314	1.2634	0.2226	0.8525	0.8616	0.9619	3.4353
ANFIS-ACO	Training	0.9161	1.5012	0.2543	0.8392	0.8797	0.9548	4.1635
	Testing	0.9312	1.2025	0.2317	0.8664	0.8995	0.9635	3.3366
Lasso-Reg	Training	0.9154	1.5070	0.2493	0.8380	0.8782	0.9543	4.1796
	Testing	0.9327	1.1907	0.2245	0.8690	0.8977	0.9641	3.3031
**C4**								
ANFIS	Training	0.9482	1.1888	0.2145	0.8992	0.9268	0.9729	3.2971
	Testing	0.9172	1.3274	0.2513	0.8372	0.9068	0.9571	3.6828
ANFIS-OA	Training	0.9392	1.2878	0.2227	0.8817	0.9202	0.9683	3.5697
	**Testing**	**0.9402**	**1.1489**	**0.2073**	**0.8780**	**0.9121**	**0.9687**	**3.1689**
ANFIS-SMA	Training	0.9168	1.4958	0.2454	0.8404	0.8735	0.9547	4.1479
	Testing	0.9335	1.1882	0.2179	0.8696	0.8887	0.9641	3.2897
ANFIS-ACO	Training	0.9226	1.4447	0.2557	0.8511	0.8901	0.9586	4.0064
	Testing	0.9367	1.1602	0.2326	0.8756	0.8851	0.9653	3.2158
Lasso-Reg	Training	0.9165	1.4977	0.2403	0.8399	0.8796	0.9550	4.1538
	Testing	0.9344	1.1781	0.2140	0.8718	0.8966	0.9650	3.2649

Significant values are in bold.

**Figure 7:**
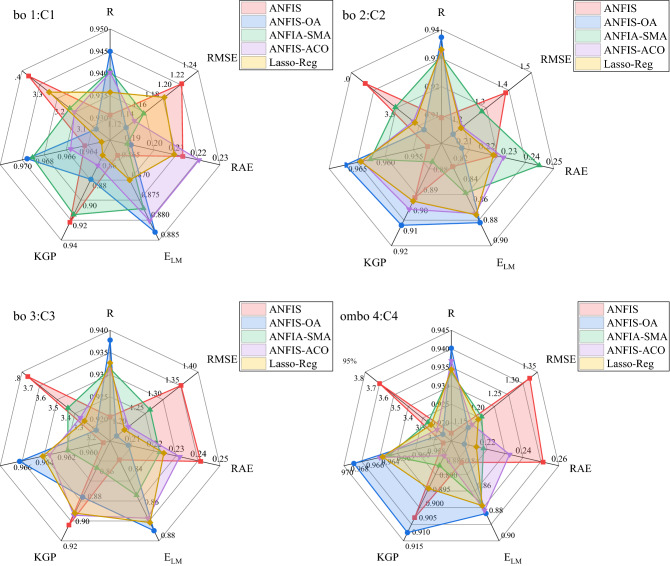
the presentation of spider plot for the developed models and input combniations.

The results were also appraised using the spider chart in Fig. 7 to compare the performance of ML models in terms of R, RMSE, RAE, E_LM_, KGE, I_A_, and U_95%_. These figures also clearly demonstrate the superiority of the hybrid ANFIS-OA model over the other ML models. Thus, the outcomes of the applied ML models in C1, C2, C3, and C4 scenarios are ranked in the following order, i.e., ANFIS-OA > ANFIS-SMA > ANFIS-ACO > Lasso-Reg > ANFIS.

**Figure 8:**
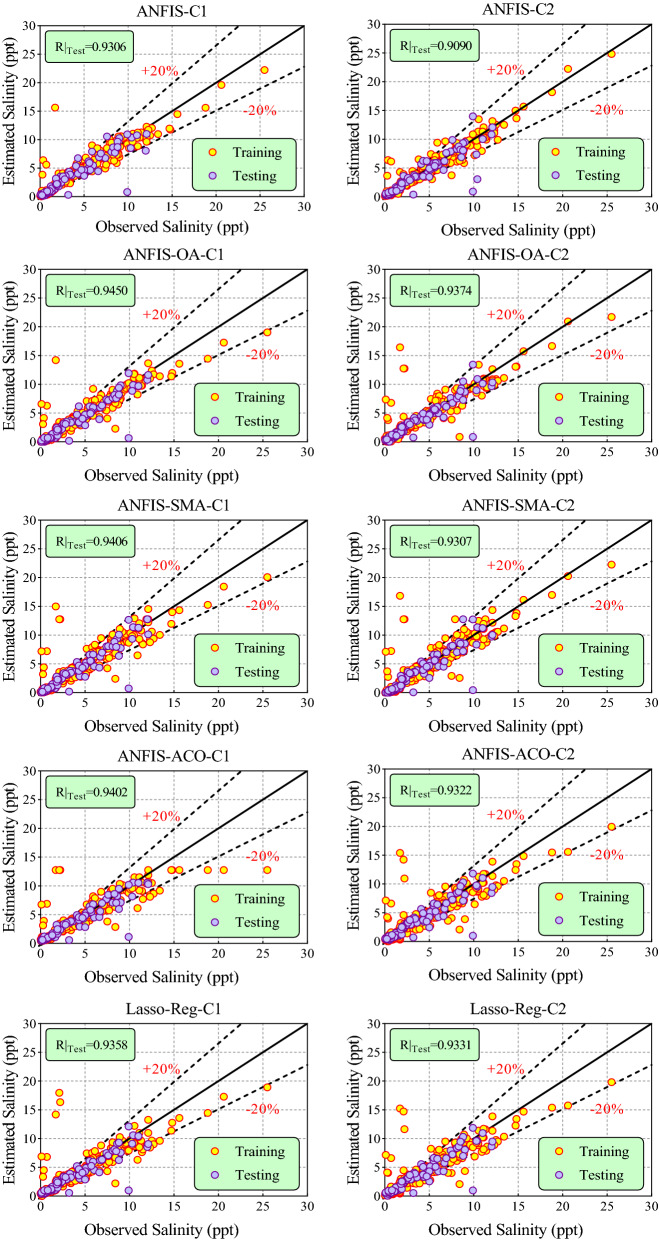
Scatter plot of observational and predicted values of salinity of groundwater using the provided models in all the scenarios.

**Figure 9:**
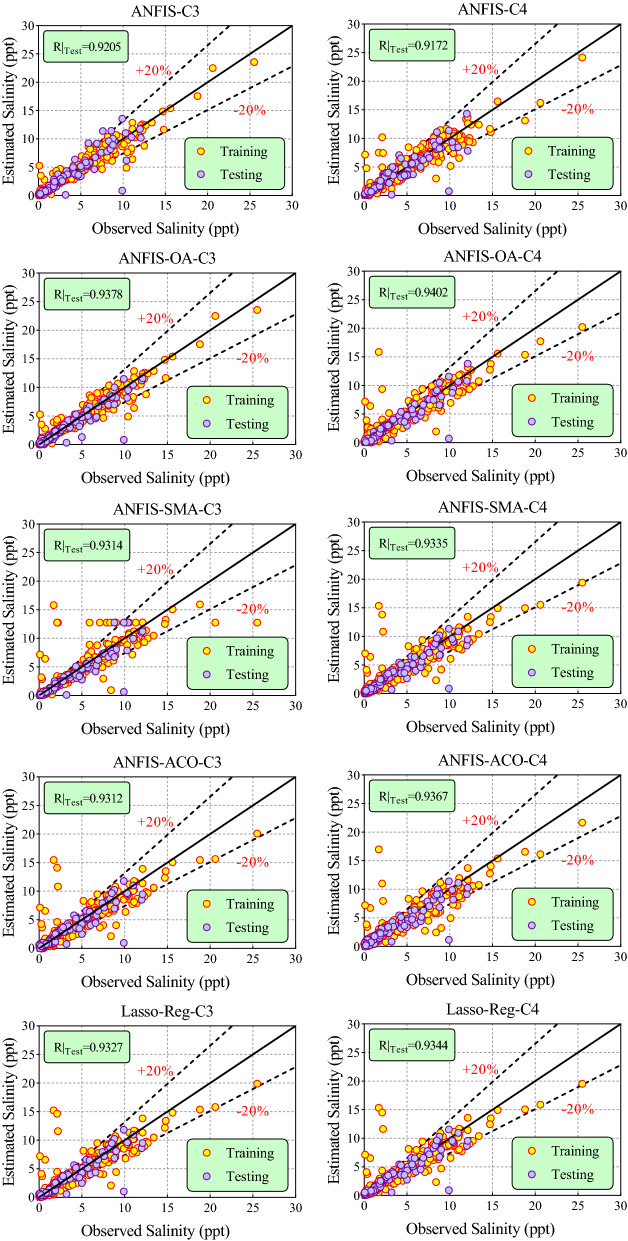
Continue; scatter plot of observational and predicted values of salinity of groundwater using the provided models in all the scenarios.

Figures 8 and 9 illustrate the scatter plots of observed versus predicted values of groundwater salinity by the ANFIS, ANFIS-OA, ANFIS-SMA, ANFIS-ACO, and Lasso-Reg models to C1, C2, C3, and C4 scenarios during training and testing phases. The outputs yielded by the ML models were plotted about the 45° (1:1 line or best fit line—solid black line) along with relative error bands of $\pm$ 20% (dash-dot lines). Another metric i.e., R (coefficient of correlation) between observed versus predicted values of groundwater salinity in testing, was displayed to assess the effectiveness of the ML models. These figures show that the ANFIS model optimized with the OA algorithm has predicted groundwater salinity values close to the observed values or most of the predicted data centered towards the 1:1 line within $\pm$ 20% error bands in all scenarios. Furthermore, the highest value of R = 0.9450, 0.9374, 0.9378, and 0.9402 was gained by the hybrid ANFIS-OA model than other ML models in C1, C2, C3, & C4 scenarios during the testing stage. Comprehensively, the ANFIS model tuned with an AO nature-inspired algorithm can be considered a robust and reliable model for predicting groundwater salinity in the study region.

## Discussion

**Figure 10:**
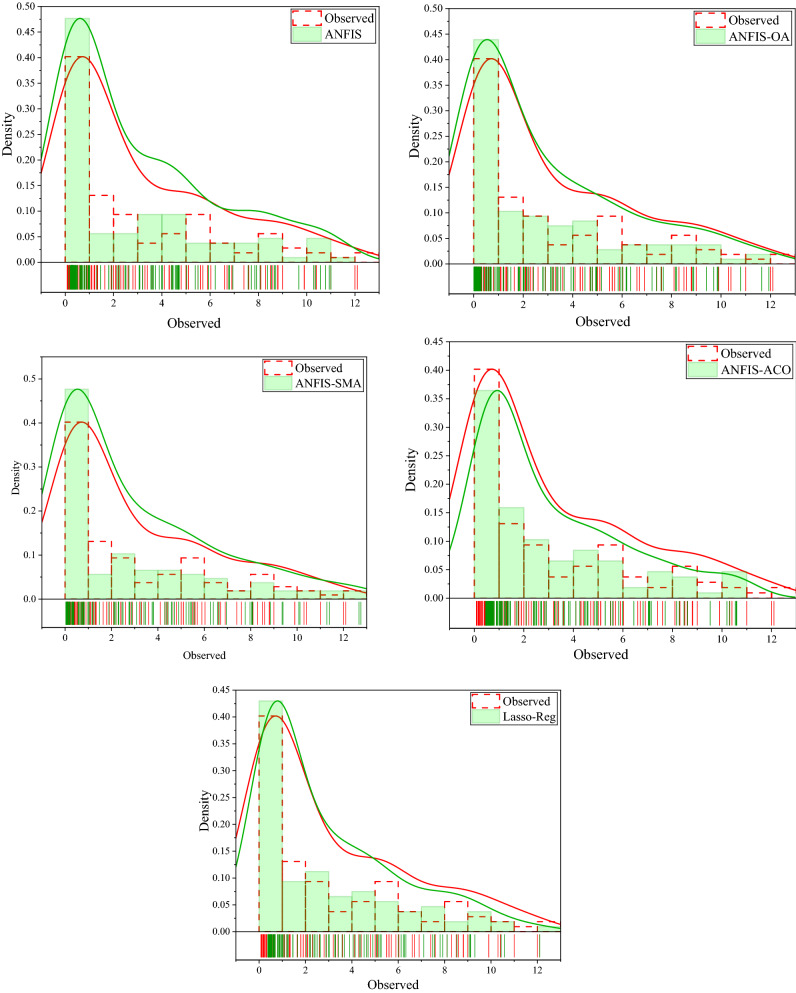
Control Rug and density distribution of Histogram Plot for all the provided models in the optimal scenario in the testing phase for predicting the salinity of groundwater.

Accurate monitoring and prediction of soil salinity are essential for sustainable development, land management, water quality, and agricultural activities, especially in arid and semi-arid regions^106^. Therefore, other criteria to examine the performance of applied hybrid and standalone ML models under different scenarios for predicting groundwater salinity are the control rug and density distribution. As mentioned in Table 4, scenario C1 was considered optimal for groundwater salinity prediction in the study area according to the comparison results. So, Fig. 10 demonstrates the control rug and density distribution histogram of ANFIS, ANFIS-OA, ANFIS-SMA, ANFIS-ACO, and Lasso-Reg models corresponding to C1 in the testing stage. These figures also confirm the supremacy of the OA algorithm over the others in groundwater salinity prediction.

**Figure 11:**
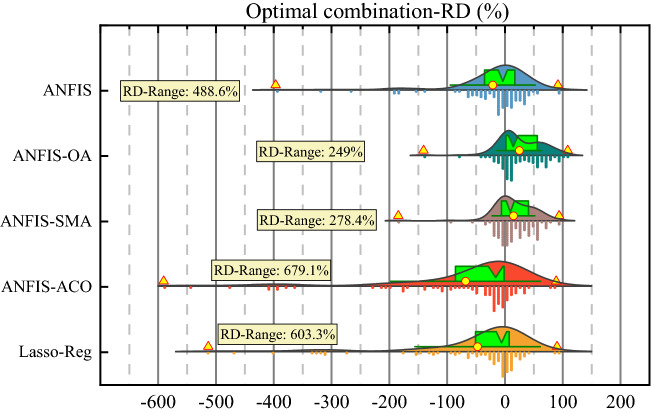
Relative devotion to the predictive model in the optimal scenario for diagnostic analysis for the testing stage.

Similarly, Fig. 11 shows the relative deviation (RD) of predicted groundwater salinity values by ANFIS, ANFIS-OA, ANFIS-SMA, ANFIS-ACO, and Lasso-Reg models for the C1 scenario during the testing period regarding the observed values. The RD was minimum in for the ANFIS-OA (249%) model than ANFIS (488.6%), ANFIS-SMA (278.4%), ANFIS-ACO (679.1%), and Lasso-Reg (603.3%) models. Because of RD%, the ML models attain ANFIS-OA > ANFIS-SMA > ANFIS > Lasso-Reg > ANFIS-ACO pattern. This RD analysis also supports the OA algorithm's effectiveness in optimizing the ANFIS model's performance in groundwater salinity prediction in the study region.

**Figure 12:**
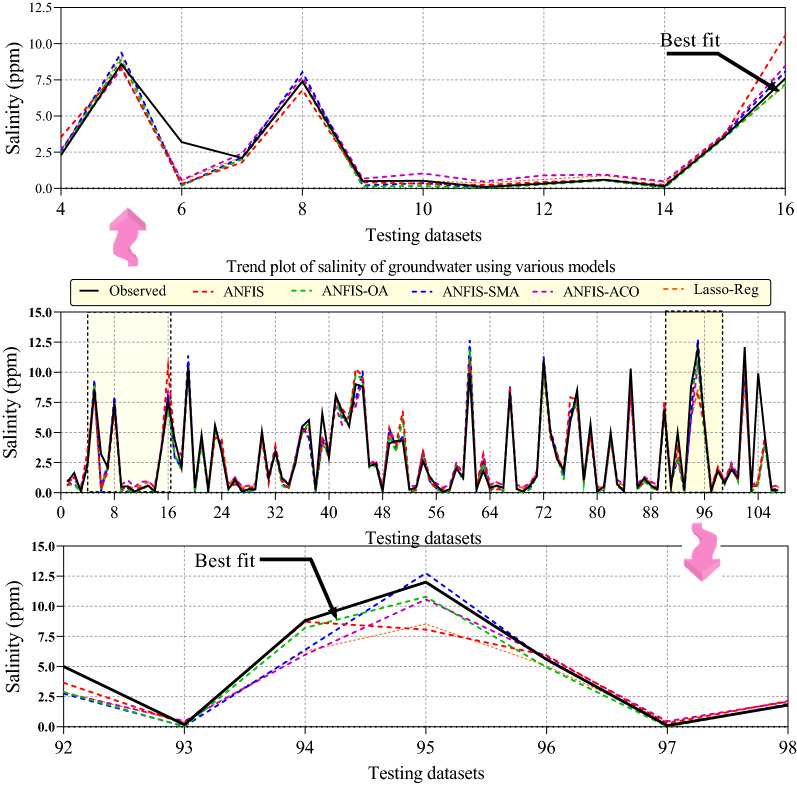
comparison between the observational and values of salinity of groundwater via the provided models in the optimal scenario.

Figure 12 displays the temporal variation of groundwater salinity predicted by the ANFIS, ANFIS-OA, ANFIS-SMA, ANFIS-ACO, and Lasso-Reg models corresponding to the C1 optimal scenario in the testing stage. The predicted values of the salinity of groundwater are distributed or plotted concerning the observed values of groundwater salinity (solid black line). The outcomes of the hybrid ANFIS-OA model (dash-dot green line) have much better matching with experimental groundwater salinity values and designate the dominance of the ANFIS-OA model.

**Figure 13:**
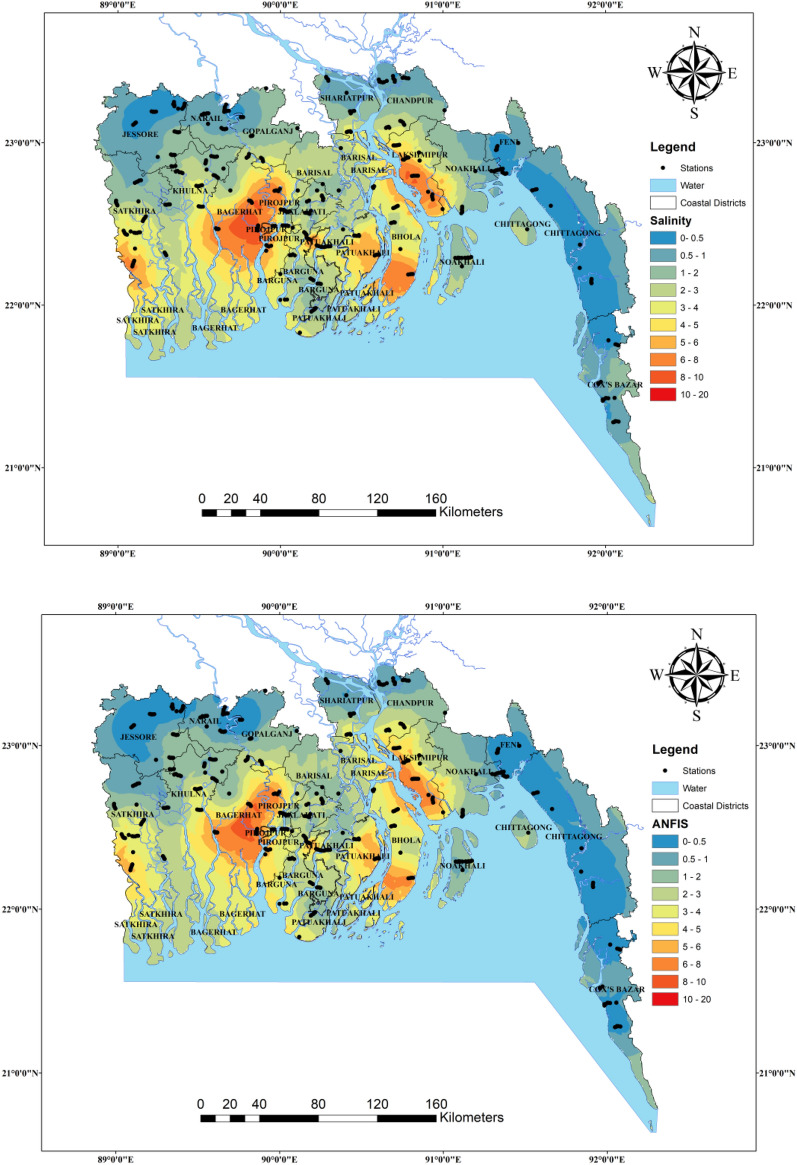

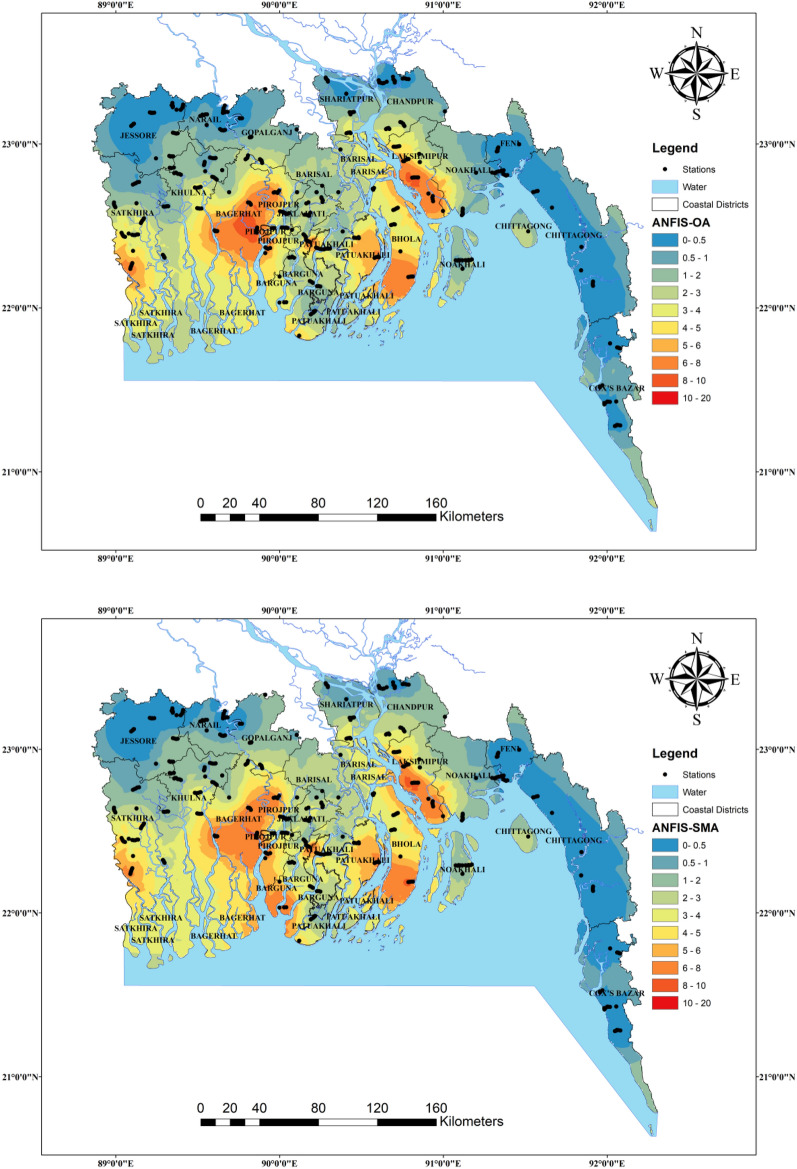

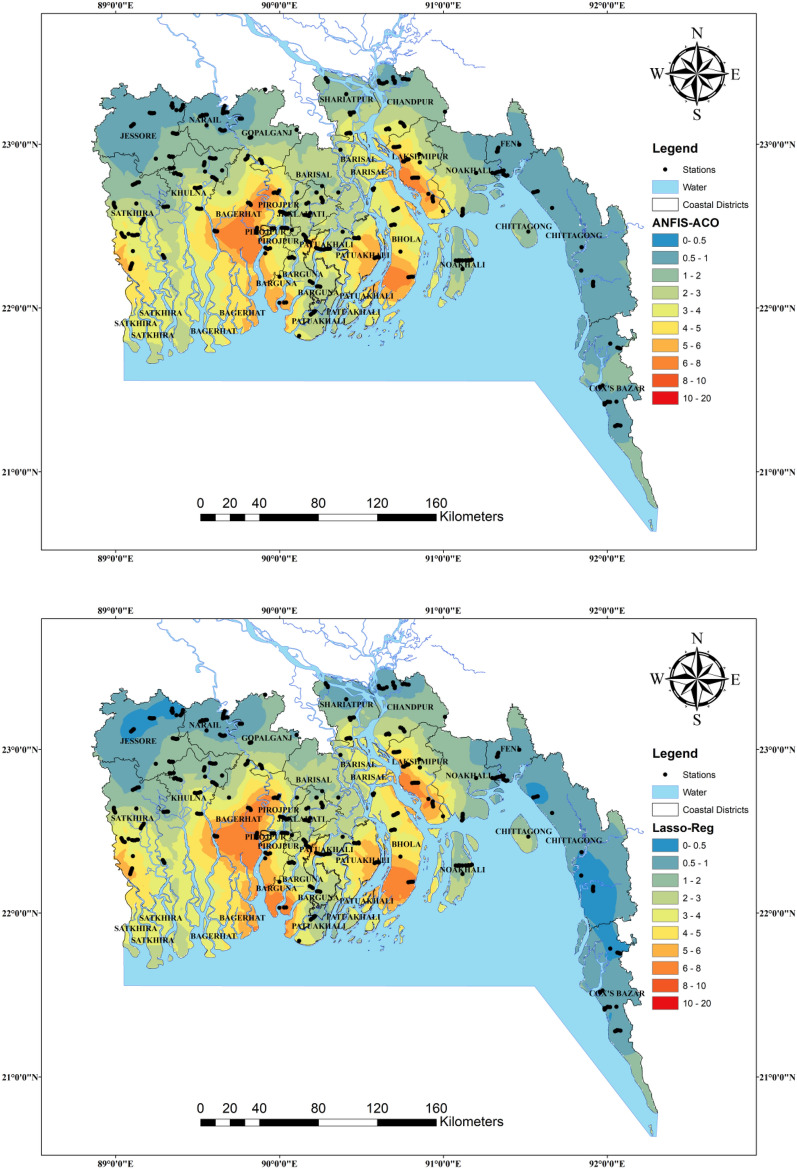
Salinity susceptibility maps for the groundwater samples collected from the coastal regions of Bangladesh.

Figure 13 shows the spatial pattern of groundwater salinity yielded by ANFIS, ANFIS-OA, ANFIS-SMA, ANFIS-ACO, and Lasso-Reg models under the C1 scenario in the testing phase concerning the observed values. The spatial interpolation was done by combining the training and testing datasets using IDW (inverse distance weighted) method. According to these spatial maps, the difference between the observed (left top figure) and the ANFIS, ANFIS-SMA, ANFIS-ACO, and Lasso-Reg models is high but much similar smooth pattern ANFIS-OA model estimates. Thus, the ANFIS with OA algorithm can distinguish the different ranges of groundwater salinity from the other ML models. Wu et al.^107^ predicted and mapped the spatial distribution of soil salinity in central Mesopotamia of Iraq by employing the support vector machine (SVM) and random forest regression (RFR) algorithms. They found that the RFR model provided better estimates than the SVM model and stated that the spatial map of soil salinity prepared by the RFR algorithm outcomes helps maintain the agricultural activities and sustainable development in the study area. In addition, the spatial distribution maps of the present study will help understand the vulnerability of salinity in groundwater on a regional scale and adopt preventive measures according to the level of salinity of groundwater.

Furthermore, to make the current research more concur and impactful, results were compared with the existed studies on soil salinity prediction using ML models in a different part of the world^108–110^. Wang et al.^111^ predicted soil salinity in three regions, i.e., Qitai, Kuqa, and Yuta oases of China, by using five ML algorithms, including MARS (multiple adaptive regression splines), CART (classification and regression trees), RF (random forest tree ensembles), SGT (stochastic gradient tree boost), and LASSO (least absolute shrinkage and selection operator). According to the results of the comparison, the SGT algorithm was found most suitable for predicting the soil salinity in three distinct regions. Ma et al.^106^ applied XGBoost (extreme gradient boosting), CART, and RF models to predict the soil salinity in the Ogan-Kuqa river oasis in China using remote sensing and topographical observations. They found that the XGBoost model achieved better prediction (R^2^ = 0.68, RMSE = 10.56 dS m^−1^) than CART (R^2^ = 0.57, RMSE = 12.20 dS m^−1^), and RF (R^2^ = 0.63, RMSE = 11.41 dS m^−1^) models. Wang et al.^112^ employed three ML algorithms, i.e., SVM, ANN, and RF, to predict the soil salinity in China's KPNR (Kongterik Pasture Nature Reserve).

Results show the better performance of SVM (R^2^ = 0.88, RMSE = 4.89 dS m^−1^) model than RF (R^2^ = 0.27, RMSE = 10.61 dS m^−1^) and ANN (R^2^ = 0.57, RMSE = 8.15 dS m^−1^) models. The literature above also established the supremacy of ML algorithms for predicting the soil salinity in different environmental conditions and strongly supports the outcomes of the present study, and endorses the application of hybrid ML models to predict the salinity of groundwater in the study region. The occurrence of major seawater intrusion in the multi-aquifers in the southwest coastal zone was indicated by extremely high salinity levels. Climate change effects such as sea-level rise, storm surges, and water logging, to the best of our knowledge, also increase salt intrusion (Na-Cl type water) along the coast. As a result, salinity-induced water is projected to move further inland, increasing contamination intensity^113^. The lack of surface freshwater supplies, such as downstream river flow, long dry periods, shrimp farming, and rainfall uncertainty, may cause changes in the coastal hydrogeologic environment, causing instability in groundwater recharge, storage, and flow. Our study presents a novel technique to aid water managers and decision-makers in safeguarding groundwater resources against saline water intrusion. Constructing accurate salinity susceptibility maps can lead to increased groundwater resource management stretegies and environmental sustainability. Comparing the current study's results with the previous similar regional investigation demonstrates that the ANFIS-AO regarding higher accuracy (R = 0.945) resulted in the promising outcomes than the Catboost model (R = 0.916) for predicting the groundwater salinity of multi-layer aquifers of Mekong Delta, Vietnam^114^ and extreme gradient boosting (EGB) model (R = 0.943) for estimating the groundwater salinity in the southern coastal aquifer of the Caspian Sea, Iran^38^.

## Conclusion and future direction

In this research, a novel Nature-inspired ANFIS model (i.e., ANFIS-AO) along with ANFIS-SMA, ANFIS-ACOR, individual ANFIS, and Lasso-Reg coastal multi-aquifers in some regions of Bangladesh based on ten water quality indices promised of depth, pH, Ca^2+^ (mg/l), Mg^2+^ (mg/l), Na^+^ (mg/l), K^+^ (mg/l), HCO_3_^−^ (mg/l), SO_4_^2−^ (mg/l), PO_4_^2−^ (mg/l), Cl^−^ (mg/l). In the pre-processing stage, the training dataset was explored using the B-RF feature selection, indicating each feature's importance degree in modeling salinity. The outcomes of pre-processing ascertained that SO_4_^2−^ (mg/l) and were neglected as the candidate inputs and Cl^−^ (mg/l), Na^+^, and Mg^2+^ (mg/l) were indicated as the most significant features. Based on the mentioned feature selecting process, four input combinations were examined to assess the compatibility of the predictive ML-based models. A careful review of the statistical criteria and graphical analysis of the employed ML models shows that the best results are related to the C1, C4, C2, and C3, respectively (C1 > C4 > C2, C3). The hybrid ANFIS-OA approach regarding the most promising metrics (R = 0.9450, RMSE = 1.1253 ppt, KGE = 0.9146, and U_95%_ = 3.0632**)** in the testing phase was superior to the ANFIS-SMA (R = 0.9406, RMSE = 1.1534 ppm, KGE = 0.8793, and U_95%_ = 3.1632), ANFIS-ACOR (R = 0.9402, RMSE = 1.1388 ppm, KGE = 0.8653, and U_95%_ = 3.1822), Lasso-Reg (R = 0.9358, RMSE = 1.1863 ppm,and KGE = 0.8552), and ANFIS (R = 0.9306, RMSE = 1.2139 ppm, and KGE = 0.9222) models. Besides, the diagnostic assessment of the superior candidate input combination (C1) ascertained that the ANFIS-OA concerning the least RD value (249%) attained the most reliable predicted salinity in coastal multi-aquifer followed by the ANFIS-SMA (278.4%), ANFIS (488.6%), ANFIS-ACO (679.1%), and Lasso-Reg (603.3%), respectively. Finally, a comparison of the spatial distribution of salinity in the aquifers of the coastal areas of Bangladesh shows well that the ANFIS-OA method has the best agreement with the actual salinity distribution, while the ANFIS-SMA method is in second place. It is worth noting that the IDW method was employed to interpolate the data points of each predictive model to provide the spatial pattern contours. Since the frameworks presented in this research are based on a robust pre-processing aim to identify the most influential input combinations, the degree of uncertainty of the models will be shallow. Besides, the hybrid neuro-fuzzy models with minor limitations can be used for other water quality indices even in other areas of study. We added this augmentation into the discussion section.

The current research was adopted to develop a hybrid version of ML models, and the research findings were successfully approached. Future research direction can be established on different aspects. For instance, studying the data, models, and input parameters uncertainties, investigating the seawater intrusion and its effects on the salinity concentration, and identifying the essential connection between groundwater salinity and corps/plantations’ health and contamination. As another alternative future study, a robust classification method can assess the salinity risks in the entire coastal multi-aquifer system. To lessen and control the deterioration of groundwater quality in the coastal zone, it is critical to avoid leaching salinity intrusion and toxic soil contents into groundwater. The study outcomes can assist the policy-makers and respective agencies in managing and protecting the water resources in the coastal region of Bangladesh. Regionalization of salinity estimation has potential implications for rationally utilizing and developing water resource strategies and plans for reducing vulnerability. The classification methods developed as a future direction can be a possible alternative for salinity assessment in any coastal plain with similar aquifer features and hydrogeologic settings.

## Data Availability

The data generated or analyzed during this study are available from the corresponding author on reasonable request.
